# 阿伐替尼治疗异基因造血干细胞移植后分子生物学阳性的伴KIT突变CBF-AML的疗效及安全性

**DOI:** 10.3760/cma.j.cn121090-20240129-00043

**Published:** 2024-08

**Authors:** 娟 王, 璎玲 祖, 瑞瑞 桂, 珍 李, 䶮莉 张, 健 周

**Affiliations:** 郑州大学附属肿瘤医院，河南省肿瘤医院血液科，郑州 450008 Department of Hematology, Henan Cancer Hospital, the Affiliated Cancer Hospital of Zhengzhou University, Zhengzhou 450008, China

**Keywords:** 核心结合因子类, 白血病，髓样，急性, 造血干细胞移植, 阿伐替尼, 基因，KIT, Core binding factors, Leukemia, myeloid, acute, Hematopoietic stem cell transplantation, Avapritinib, Gene, KIT

## Abstract

**目的:**

探讨阿伐替尼治疗异基因造血干细胞移植（allo-HSCT）后分子生物学阳性的伴KIT突变核心结合因子相关性急性髓系白血病（CBF-AML）的疗效及安全性。

**方法:**

纳入河南省肿瘤医院2021年12月至2023年3月收治的6例allo-HSCT后分子生物学阳性的伴KIT突变CBF-AML患者，均接受阿伐替尼治疗，回顾性分析阿伐替尼的疗效及安全性。

**结果:**

阿伐替尼治疗1个月后，6例患者融合基因转录水平均较前下降，其中5例患者转录水平下降≥1 log。4例患者接受阿伐替尼治疗时间≥3个月，4例患者融合基因均转阴，转阴的中位时间为2.0（1.0～3.0）个月。至随访截止，4例患者无复发生存。最常见的不良反应是骨髓抑制，包括中性粒细胞减少2例、血小板减少2例、贫血1例。非血液学不良反应包括恶心2例、水肿1例、记忆力减退1例，均为1～2级。

**结论:**

阿伐替尼可作为allo-HSCT后分子生物学阳性的伴KIT突变CBF-AML患者的有效治疗手段，主要的不良反应为骨髓抑制，患者耐受性良好。

## Abstract (en)

**Objective:**

To investigate the efficacy and safety of avapritinib in the treatment of molecular biologically positive core binding factor-acute myeloid leukemia（CBF-AML）with KIT mutation after allogeneic hematopoietic stem cell transplantation（allo-HSCT）.

**Methods:**

We retrospectively analyzed the clinical data of six patients with molecular biologically positive CBF-AML with KIT mutation after allo-HSCT, who were treated with avapritinib at Henan Cancer Hospital from December 2021 to March 2023, and evaluated the efficacy and safety of avapritinib.

**Results:**

After 1 month of treatment with avapritinib, the transcription level of the fusion gene decreased in six patients, and the transcription level decreased by ≥1 log in five patients. In four patients who received avapritinib for ≥3 months, the fusion gene turned negative, and the median time to turn negative was 2.0（range: 1.0–3.0）months. Up to the end of follow-up, four patients had no recurrence. The most common adverse reaction of avapritinib was myelosuppression, including neutropenia in two cases, thrombocytopenia in two cases, and anemia in one case. The non-hematological adverse reactions were nausea in two cases, edema in one case, and memory loss in one case, all of which were grades 1–2.

**Conclusion:**

Avapritinib was effective for molecular biologically positive CBF-AML patients with KIT mutation after allo-HSCT. The main adverse reaction was myelosuppression, which could generally be tolerated.

核心结合因子相关性急性髓系白血病（core binding factor-acute myeloid leukemia, CBF-AML）约占AML的15％[1]–[2]，是指以染色体t（8;21）（q22;q22）或inv（16）（p13;q22）/t（16;16）（p13;q22）异常为特征的白血病亚型，染色体易位重排导致融合基因RUNX1::RUNX1T1（AML1/ETO）和CBFB::MYH11的形成[3]–[4]。与其他亚型白血病相比，CBF-AML患者预后较好，但仍有约40％的患者因复发无法获得长期生存[5]。异基因造血干细胞移植（allo-HSCT）可降低CBF-AML患者复发率，提高生存率[6]–[8]。然而，仍有约20％的CBF-AML患者移植后复发[9]–[11]。随着二代测序技术的广泛应用，研究者发现CBF-AML患者常伴基因突变，甚至合并多个基因突变。其中，KIT突变存在于约40％的t（8;21）异常AML和33％的inv（16）异常AML患者中[12]，影响CBF-AML患者的预后。

除常规化疗外，伴KIT突变CBF-AML患者可联合小分子激酶抑制剂，如伊马替尼、达沙替尼、米哚妥林等，但部分患者因无法耐受不良反应而停药。阿伐替尼是一种强效、高选择性KIT和血小板衍生生长因子受体α（Platelet-derived growth factor receptor α，PDGFRA）抑制剂，目前在伴KIT突变CBF-AML患者中应用较少[13]–[15]，在移植后分子生物学阳性患者中应用的经验更少。因此，本研究通过收集应用阿伐替尼治疗的allo-HSCT后分子生物学阳性伴KIT突变CBF-AML患者的临床资料，评估阿伐替尼的疗效和安全性，为患者的治疗提供参考。

## 病例与方法

1. 病例：本研究纳入2021年12月至2023年3月于河南省肿瘤医院接受allo-HSCT的KIT突变阳性CBF-AML患者12例，其中移植后分子生物学阳性、接受阿伐替尼治疗且资料完整的患者6例，收集上述6例患者的临床资料，包括年龄、性别等一般情况，初诊时血常规、骨髓形态学及流式细胞术原始细胞比例、融合基因定性定量、KIT突变类型及化疗疗效等；移植后定期检测融合基因定量及突变频率，并进行疗效及预后评估。分子生物学阳性包括分子生物学复发及分子生物学持续阳性，前者指骨髓实时荧光定量聚合酶链反应（RQ-PCR）检测融合基因由阴性转为阳性；后者指连续3次及以上融合基因定量检测阳性，两者均未达到形态学复发标准且无髓外复发。血液学复发定义为融合基因阳性，并经复查证实骨髓形态学复发或发生髓外白血病。

2. 移植前情况及移植方案：移植前所有患者均接受诱导化疗，完全缓解（CR）后巩固治疗至少2个疗程。5例患者移植时为第1次CR期，1例患者移植时疾病未缓解。所有患者均采用FLUBuMelMe-CCNU（氟达拉滨+白消安+美法仑+司莫司汀）方案，其中未缓解患者应用CLAG（克拉屈滨+阿糖胞苷+粒细胞集落刺激因子）方案序贯预处理。无关供者移植在此基础上加用抗胸腺细胞球蛋白（ATG）。亲缘供者移植在回输后第5天用ATG，所有患者均应用移植后环磷酰胺（Posttransplant cyclophosphamide，PTCY）预防移植物抗宿主病（GVHD），同胞全相合造血干细胞移植患者采用环孢素A（CsA）或他克莫司（FK506）联合短程吗替麦考酚酯预防GVHD，单倍型造血干细胞移植及无关供者移植患者在此基础上联合芦可替尼。移植物为G-CSF动员的外周血干细胞。

3. 融合基因：融合基因定量检测采用RQ-PCR法，内参基因选用ABL基因，融合基因转录水平=融合基因转录子拷贝数/ABL转录子拷贝数×100％。在预处理前及移植后1、2、3、4.5、6个月进行融合基因定量检测，随后每3个月检测1次。若应用阿伐替尼，则每月检测1次，直至阿伐替尼停用后3个月，随后根据融合基因水平可每3个月检测1次。

4. 治疗：阿伐替尼的初始应用剂量：体重<50 kg剂量为50 mg/d，体重≥50 kg剂量为100 mg/d，应用阿伐替尼后1个月内每周检测血常规、肝功能、肾功能、心肌酶、电解质等，若无不良反应，2个月后可每2周检测1次血常规等。若出现3级以上不良反应可停药，恢复至基线水平后可减量应用。阿伐替尼持续应用直至出现不可耐受的不良反应、疾病进展、死亡或医师决定停药。

5. 随访：采用门诊和电话随访。随访截止日期为2023年12月31日，中位随访时间为7.0（4.0～8.0）个月。

6. 统计学处理：计量资料采用中位数（范围）描述，计数资料采用例数（％）描述。

## 结果

1. 一般资料：接受阿伐替尼治疗的6例患者中，男2例，女4例，中位年龄37（6～57）岁，4例融合基因类型为RUNX1::RUNX1T1，2例为CBFB::MYH11。6例患者的KIT突变可分为两类：5例为单一D816（3例D816V、1例D816Y、1例D816V和D816Y双阳），1例为Asp419del。所有患者在1或2个周期的诱导化疗后达CR，1例患者allo-HSCT前复发。allo-HSCT前融合基因转录水平的中位数为3.29％（0.05％～114.39％），6例患者初诊时血常规、骨髓形态学及流式细胞术原始细胞比例、融合基因类型、KIT突变类型见表1。6例患者移植前状态、移植前融合基因水平及移植前KIT突变频率等情况见表2。2例患者行同胞全相合造血干细胞移植，2例行单倍体造血干细胞移植，2例行HLA配型相合无关供者造血干细胞移植。回输单个核细胞和CD34^+^细胞的中位数分别为10.8（8.1～13.7）×10^8^/kg和6.6（4.2～9.9）×10^6^/kg。粒细胞和血小板植入的中位时间分别为12（10～15）d和11（9～17）d。

**表1:** 6例核心结合因子相关性急性髓系白血病患者的基线资料

例号	性别	年龄（岁）	初诊时血常规			初诊时原始细胞比例（％）		融合基因类型	KIT突变类型
			WBC（×10^9^/L）	HGB（g/L）	PLT（×10^9^/L）	骨髓形态学	流式细胞术		
1	女	57	5.24	76	5	55.60	68.00	RUNX1::RUNX1T1	D816V
2	女	26	8.55	93	26	50.40	54.20	RUNX1::RUNX1T1	D816V、D816Y
3	女	48	12.10	72	16	80.40	30.80	RUNX1::RUNX1T1	D816Y
4	女	12	49.91	60	7	22.00	59.49	RUNX1::RUNX1T1	p.Asp419del
5	男	54	173.64	66	10	75.70	83.61	CBFB::MYH11	D816V
6	男	6	21.34	63	12	69.00	22.50	CBFB::MYH11	D816V

**表2:** 6例核心结合因子相关性急性髓系白血病患者移植前及移植情况

例号	移植前状态	移植前融合基因水平（%）	移植前KIT突变频率（%）	供者类型	回输MNC（×10^8^/kg）	回输CD34^+^细胞（×10^6^/kg）	粒细胞植入时间	血小板植入时间
1	CR	39.63	5.360	无关	9.98	4.19	10 d	9 d
2	CR	0.77	0.016、0.090	无关	8.68	7.90	12 d	10 d
3	CR	5.60	阴性	同胞全相合	11.69	5.03	10 d	10 d
4	CR	0.97	阴性	同胞全相合	8.10	5.20	14 d	14 d
5	复发	114.39	11.200	单倍体	12.29	9.94	15 d	17 d
6	CR	0.05	阴性	单倍体	13.70	9.50	12 d	11 d

**注** CR：完全缓解；MNC：单个核细胞

2. 疗效：4例患者分子生物学阳性后减停免疫抑制剂，接受供者淋巴细胞回输（DLI）、地西他滨、维奈克拉治疗，其中3例接受过达沙替尼治疗，上述治疗无效后应用阿伐替尼。另2例患者分子生物学阳性后直接应用阿伐替尼，免疫抑制剂减量。所有患者阿伐替尼治疗期间未出现GVHD。开始应用阿伐替尼的中位时间为移植后7.3（1.0～18.0）个月，持续应用阿伐替尼的中位时间为5.0（1.0～8.0）个月。阿伐替尼治疗前融合基因转录水平的中位数为4.71％（1.34％～7.86％），阿伐替尼治疗1个月后，融合基因转录水平的中位数为0.05％（0～0.80％），较前明显下降。

与治疗前转录水平相比，阿伐替尼治疗1个月后6例患者融合基因转录水平均较前下降，其中5例患者转录水平下降≥1 log，此5例患者均为D816突变，其中1例患者转录水平下降≥2 log，1例患者融合基因转阴，该例融合基因转阴患者因经济原因治疗1个月后停药，停药后融合基因持续阴性。仅1例患者接受阿伐替尼治疗1个月后RUNX1::RUNX1T1融合基因转录水平下降<1 log，该患者为Asp419del突变，在治疗2个月后融合基因转录水平较治疗前升高10倍，治疗无效停药，停药1个月后出现血液学复发，最终因疾病进展死亡。

4例患者接受阿伐替尼治疗的时间≥3个月，4例患者融合基因均转为阴性，转阴的中位时间为2.0（1.0～3.0）个月。其中1例患者在阿伐替尼治疗3个月后因3级骨髓抑制停药，停药后融合基因持续阴性。1例患者在阿伐替尼治疗4个月后因2级骨髓抑制减量，减量3个月后融合基因复阳，再次加至原始剂量后融合基因转阴。1例患者在阿伐替尼治疗7个月后因合并新型冠状病毒肺炎而停药，最终因肺部感染死亡。2例患者目前仍在接受阿伐替尼治疗。

从开始阿伐替尼治疗至随访截止，中位观察时间为7.0（4.0～8.0）个月，5例患者融合基因转阴，融合基因转阴的中位时间为2.0（1.0～3.0）个月。4例RUNX1::RUNX1T1阳性患者中，3例患者融合基因转阴，转阴时间均为阿伐替尼治疗后2个月。2例CBFB::MYH11阳性患者融合基因均转阴，分别在阿伐替尼治疗后1个月和3个月。1例患者接受阿伐替尼治疗2个月后因无效停药，停药后1个月出现血液学复发，最终因疾病进展死亡，1例患者在阿伐替尼治疗7个月后因合并新型冠状病毒肺炎而停药，最终因肺部感染死亡。至随访截止，共4例患者无复发生存。

3. 不良反应：2例患者出现血液学不良反应，1例在阿伐替尼治疗3个月后出现3级中性粒细胞减少、贫血、血小板减少，导致治疗中断，停药后骨髓抑制恢复至1级，融合基因持续阴性；1例患者在阿伐替尼治疗4个月后出现2级中性粒细胞减少、1级血小板减少，阿伐替尼减量为50 mg/d后血象恢复正常，减量3个月后融合基因复阳，再次加至原始剂量后融合基因转阴，仍出现1级中性粒细胞减少。非血液学不良反应为恶心2例、水肿1例、记忆力减退1例，均为1～2级，记忆力减退患者在停药后记忆力恢复正常。

## 讨论

目前移植后复发仍是影响AML患者长期生存的关键问题，其中KIT突变是CBF-AML患者移植后复发的高危因素[16]。尽管预防性应用干扰素α和DLI，2年累积复发率仍分别为55.6％和31.4％[8]。移植后较高的融合基因转录水平可以准确预测复发[9],[16]。因此，当出现移植后分子生物学阳性时，应及时采取措施，降低疾病复发风险。在本研究中，移植后出现分子生物学阳性时及时应用阿伐替尼治疗，仅1例患者因阿伐替尼无效停药，停药1个月后血液学复发，最终因疾病进展死亡。其余5例患者融合基因均转阴，至随访截止，共4例患者无复发生存。

伴KIT突变CBF-AML患者目前主要的靶向治疗药物为小分子激酶抑制剂，一些靶向FLT3和ABL等酪氨酸激酶受体的药物已被证实对KIT突变具有一定的疗效[17]–[19]。一项伊马替尼联合再诱导化疗治疗复发/难治性KIT突变AML的Ⅰ/Ⅱ期研究结果显示，约62％的患者获得CR[20]。达沙替尼也是一种潜在的靶向KIT突变的药物，CALGB的单臂研究显示，接受达沙替尼联合HDAC方案治疗的CBF-AML患者中，携带KIT突变患者的无病生存期和总生存期与野生型KIT患者相当[21]。米瑞华等[22]报道的研究显示，10例伴KIT突变AML-M_2b_患者应用酪氨酸激酶抑制剂联合化疗的总有效率为80％。米哚妥林是第一代FLT3抑制剂，据报道，Ba/F3细胞中表达的KIT-D816V受体对米哚妥林敏感[23]。阿伐替尼是一种针对KIT突变的新型酪氨酸激酶抑制剂，对KIT和PDGFRA的活化环区域突变具有选择性活性，可以与细胞内结构域的活性激酶构象结合，从而抑制激酶活化，具有ATP竞争性，靶向激酶的ATP结合位点，抑制肿瘤信号转导[24]。Weisberg等[25]应用阿伐替尼与数个FLT3抑制剂进行体外药敏实验，发现阿伐替尼作用于Ba/F3 KIT-D816V细胞的效力是米哚妥林的10倍左右。

阿伐替尼起效迅速，在本研究中，6例患者在给药1个月后融合基因转录水平均下降，其中5例患者转录水平下降≥1 log，1例患者实现融合基因转阴。随着治疗时间的延长，阿伐替尼的有效率逐渐增加。接受阿伐替尼治疗≥3个月的4例患者融合基因均转阴。从开始应用阿伐替尼治疗至随访截止，中位观察时间为7.0（4.0～8.0）个月。5例患者融合基因转阴，从开始应用阿伐替尼治疗至融合基因转阴的中位时间为2.0（1.0～3.0）个月。Kong等[13]的回顾性研究报道了20例t（8;21）异常AML伴KIT突变患者，接受allo-HSCT后微小残留病阳性，接受阿伐替尼治疗，12例（60％）患者治疗1个月后RUNX1::RUNX1T1下降≥1 log，接受阿伐替尼治疗≥3个月的13例患者RUNX1::RUNX1T1均下降≥1 log。Xue等[15]报道了1例t（8;21）异常AML伴KIT D816I突变患者，在allo-HSCT复发后应用阿伐替尼治疗，1个月后RUNX1::RUNX1T1转录水平从109.4％下降到0.13％，1.5个月后融合基因转阴。白莲等[26]报道了2例伴KIT突变复发难治AML患者，1例为移植后复发，应用阿伐替尼联合维奈克拉后获得形态学无白血病状态；另1例化疗后复发，应用阿伐替尼联合维奈克拉，桥接allo-HSCT后再次获得CR。

KIT突变位点不同，患者的预后和治疗效果亦存在明显差异。Cairoli等[27]的研究报道，KIT D816突变患者的复发率高于其他KIT突变型和野生型患者（90.0％对57.1％对35.3％），2年总生存率仅为25.0％，明显低于野生型KIT患者（76.5％）。Yui等[28]也得出类似的研究结论，CBF-AML中KIT D816突变者的无复发生存率显著低于KIT N822K突变和KIT野生型患者（22.0％对53.8％对56.7％，*P*＝0.002），总生存率有同样的趋势（38.5％对77.8％对77.2％，*P*<0.001）。随着KIT突变靶向药物的应用，伴KIT突变CBF-AML患者的疗效及预后可能会有所改善。Kong等[13]的研究发现，KIT D816突变患者应用阿伐替尼的总有效率与非D816突变患者相比，差异无统计学意义。本研究中所有D816突变患者在阿伐替尼治疗1个月后融合基因转录水平均下降≥1 log。突变位点为Asp419del的患者应用阿伐替尼治疗1个月后RUNX1::RUNX1T1融合基因转录水平下降<1 log，治疗2个月后融合基因转录水平较治疗前升高10倍，停药1个月后血液学复发，最终因疾病进展死亡。

不同融合基因类型（RUNX1::RUNX1T1/CBFB::MYH11）是否影响阿伐替尼的疗效目前也不清楚。本研究中，3例患者RUNX1::RUNX1T1基因转阴，转阴时间为阿伐替尼治疗后2个月。另2例患者CBFB::MYH11融合基因均转阴，转阴时间分别为阿伐替尼治疗后1个月和3个月。

对于伴KIT突变的CBF-AML患者，阿伐替尼治疗应维持多长时间尚无定论。本研究中，1例患者在治疗1个月后CBFB::MYH11基因转阴，由于经济原因停用了阿伐替尼，至随访截止，融合基因已持续阴性6个月。另1例RUNX1::RUNX1T1阳性患者在阿伐替尼治疗2个月后融合基因转为阴性，3个月后因3级骨髓抑制而停用阿伐替尼，至随访截止，融合基因已持续阴性6个月。另1例患者在阿伐替尼治疗2个月后RUNX1::RUNX1T1转阴，治疗4个月后因2级骨髓抑制而降低剂量，RUNX1::RUNX1T1基因在阿伐替尼减量3个月后转为阳性，加量至原始剂量后1个月融合基因再次转阴。

对于allo-HSCT后接受阿伐替尼治疗的患者，最常见的不良反应是骨髓抑制，1例患者发生3级中性粒细胞减少、贫血、血小板减少，停药后恢复至1级。1例患者发生2级中性粒细胞减少、1级血小板减少。非血液学不良反应包括恶心2例、水肿1例、记忆力减退1例，均为1～2级。记忆力减退患者停药后记忆力恢复正常。Yin等[14]的研究报道，在阿伐替尼联合其他化疗药物治疗期间，4例患者中，发生3级中性粒细胞减少2例，3级贫血2例，4级血小板减少2例。Kong等[13]报道，allo-HSCT后接受阿伐替尼治疗的患者中，3例发生≥3级中性粒细胞减少，2例发生≥3级贫血，7例发生≥3级血小板减少，停药后均可恢复。

本研究存在很多不足，首先，本研究是一项回顾性研究，选择接受阿伐替尼治疗的患者时可能存在一定的偏倚。其次，本研究样本量较少，缺乏对照组。最后，本研究随访时间短，无法评估阿伐替尼治疗的长期疗效和维持时间。本研究组后期将扩大样本量，进行前瞻性多中心临床试验研究，进一步评估阿伐替尼的疗效及安全性。

综上所述，我们的研究结果表明，阿伐替尼可作为allo-HSCT后分子生物学阳性伴KIT突变CBF-AML患者的有效治疗手段，主要的不良反应为血液学不良反应，患者耐受性良好。本研究为单中心、回顾性研究且病例较少，以上结论尚需多中心、大样本量研究加以验证。
